# Practical Notes and Formulæ

**Published:** 1879-03-20

**Authors:** 


### PRACTICAL NOTES AND FORMULÆ.

**Pruritus Vulvæ.**—Dr. Mendenhall (Obstetric Gazette) says: Upon examination found the external genitals somewhat swollen and covered with minute vesicles; the inner side of the labia, urethra and entrance to vagina extremely red and tender; did not examine with the speculum. There was a slight leucorrhœa. I prescribed as follows:

℞. Soda borate pulv....................... ʒj.

Plumbi acet........................... ʒ ss.

Tr. opii................................ ʒi.

Aqua distil............................ ℥ viii. M. ft. sol.

S. When used dilute with one-fourth the quantity necessary of warm water. Apply freely on cloths saturated with this solution to the external parts affected, and also between the labia—this to be repeated two or three times every fifteen or twenty minutes. Then inject one ounce of solution into the vagina with a common glass syringe. This greatly ameliorated her sufferings. These applications were immediately followed by a free application of:

℞. Glycerin.................................... ℥ i.

Acid carbolic...............................gtt. xx M.

The relief was immediate and complete. The pruritus returned subsequently in a very light form twice, but in each instance relieved immediately by the same remedies.

Athill recommends in such cases that the patient, after she has syringed or sponged herself with warm water, lay inside the labia pieces of lint soaked in a lotion composed of carbolic acid, grs. x.; acet. morphia, viii.; dilute hydrocyanic acid, ʒii.; glycerin, ʒiv., and water ℥iv. M.

**Vaginismus.**—Vaginismus is a name given by Dr. Marion Sims to spasm of the sphincter muscle of the vagina. It seems sometimes to be a neurosis without visible cause, but may result from herpes, vaginal fissure, mucous patches, disease of urethra, and hypertrophied and painful papillæ. These various conditions require appropriate treatment. In case of vaginal fissure, Dr. Bouchert, of Paris, recommends the following:

℞. Extracti krameria (rhatany).......... ℥ ss.

Butyri cacaonis................................ ℥ i. M.

Make twelve suppositories. One to be introduced into the vagina night and morning. Prof. Carl directs to bathe the external genitals cautiously with lead-water, and afterward, when the redness has subsided, pencil the sensitive parts with:

℞. Argenti nitratis.......................... ʒ ijss.

Aquæ distil............................... ℥ i. M.

Or with:

℞. Acidi carbolic.......................grs. x.

Aquæ..................................... ʒ j.

**Lotion in Pityriasis of the Face.**—

℞. Liq. plumbi subacetatis........................f. ℥ ss.
Vitelli ovorum duorum.
Aquæ sambuci (Ph. Lond.)...........Oj. M.

**Spirit of Walnut for Vomiting.**—Ed. Mackey, M.D., M.R.C.P. (London Practitioner), recommends the spirit of walnut, made after the following formula, for vomiting:

| | | |
|---|---|---|
| ℞. | Fresh walnuts | ℥ xxx. |
| | Spirit of wine (rect.) | ℥ xij. |
| | Water, q. s. distil. | ℥ xvj. |

He says: "My own experience is, in general terms, of quick and marked benefit from drachm doses, given every one to four hours, in a little water, for hysterical vomiting, for that of obstinate dyspepsia, and of pregnancy, for anomalous cases, and even for cerebral vomiting. I have tried it in septicæmia, and cannot be surprised if in that case I was disappointed; in the other cases it has always given more or less relief.—*Louisville Medical News.*

**Asclepias in Scrofula.**—There are, I think, very few cases of scrofula that will not be greatly benefited by a persevering use of asclepias; and when combined with phytolacca decandra, I know of no prescription comparable to it in this disease, aided by malt or cod-liver oil when indicated. Especially is this true with the disease as it appears in the negro, on whom it seems to act with peculiar efficacy. My favorite formula is the following:

| | | | |
|---|---|---|---|
| ℞. | Strong decoction asclepias syriaca | ℥ xij. | |
| | Decoction phytolacca decandra | ℥ iv. | |
| | Pure whisky | ℥ vj. | |
| | White sugar | ℥ iv. | M. |

From one-half teaspoonful to two teaspoonsful thrice daily, according to age and effect produced.—DR. THOMAS *in Louisville Med. News.*

**Bronchitis.**—In bronchitis, where the cough is deep, hard and dry, use the following cough mixture:

| | | | |
|---|---|---|---|
| ℞. | Ammoniæ carbonatis | grs. xx. | |
| | Spirits of nitre | ʒ iij. | |
| | Syrupi tolu | ℥ j. | |
| | Water | ℥ iss. | M. |

Dose, one to two teaspoonsful every two to three hours.

If the patient does not rest well at night, omit one dose toward bedtime, and give fifteen to twenty grains bromide of ammonium, and of course do not awake him to give cough medicine. Another good formula is:

| | | |
|---|---|---|
| ℞. | Carb. ammoniæ | ʒ ss. |
| | Syrupi senegæ | ℥ ss. |
| | Syrupi tolu | ℥ i. |
| | Aquæ | ℥ ijss. |

Dose, one teaspoonful every three hours.—(NAPHEY.)

**For Pleurodynia.**—Irritate the chest externally with chloroform, and give internally:

| | | |
|---|---|---|
| ℞. | Sulph. quinine | |
| | Pulv. doveri | aa gr. v. |

Repeat in two hours if not relieved. Patient should go to bed and put hot rock to the feet.

**Injection for Gonorrhœa.**—Dr. M. A. Wilson, of this city, gives the following as his favorite prescription for gonorrhœa. He says it is most beneficial in the subacute stages, either recent or long standing, but not of much service in gleet. All directions in regard to urethral hygiene, usually ordered in the treatment of this obstinate affection, are of course to be insisted upon:

℞ Zinci iodid.................................. grs. v

Bismuthi subnit.......................... ℈ ij

Mucil. gum acac........................ ʒ iss

Aqua dist.................................. q. s. ad. ℥ iij

To be well shaken.

M. S.—To be injected after each urination.

This is the strength most generally serviceable, but may be varied according to the judgment of the prescriber.—*Med. Record.*

**Ergotin Suppositories.**—The following formula is that very generally used by practitioners in Ireland:

℞ Hard soap.................................. ʒ j

Water.......................................... ♏xxx

Ergotin ...................................... gr. xxxij

Glycerin.................................... ʒ ss

Dissolve the soap in the water, with a gentle heat, and add the glycerin; evaporate, to get rid of the water, add the ergotin, and pour into moulds. By this manipulation a nice suppository is obtained, which is difficult to make with glycerin alone.—*Med. and Surg. Rep.*

**Chapped Hands.**—Dr. M. A. Wilson, of this city, gives the following prescription for chapped hands:

℞ Acid carbol.................................. gr. xv

Yolk of egg.................................. one

Glycerinæ.................................. ℥ iij

A small portion to be gently smeared over the affected surface several times daily.

The wearing of a pair of cotton or old kid gloves will much assist the recovery. The hands to be kept much as possible out of water. This mixture does not "spoil" by keeping.—*Med. Recorder.*

**For Impotency.**—Dr. L. G. Lincecum writes:

℞ Phosphuret Zinci........................., grs. v
Ext. cannibis indica..................... " iv
Ext. nux vomica......................... " vii
Sul hydrastin.............................. " xxx
M. et fiat pil No. 30.

One three times a day.

**Laxative and Anodine Pill.**—

℞ Pill hydrarg.

Ext. colocynth ........................aa gr. xL

Ext. hyosciamus......................... gr. xx M.

Fiat pills No. 20.

Dose. one pill at bed time. Will tend to promote rest at night and will act mildly next day, touching the liver and unlocking the upper bowels.

**Catarrhal Fever.**—In catarrhal fever, or those forms of bronchitis attended with febrile exascerbations, the following is excellent:

℞. Sulph. quinæ....................................

Carb. ammoniæ..............................aa ʒ ss.

Syrup. senegæ...............................

Syr. accaciæ ..............................aa ℥ j.

Shake and give a teaspoonful every three or four hours. A medical friend informs us he has had best success in the influenza which has prevailed lately with the following:

℞. Pulv. doveri.....................................
Pulv. capsicum.................................aa gr. 30.

Triturate thoroughly and make ten powders. Take one every three to four hours, suspending for a time if the opiate influence should become decided. We suggest that a little quinine added to this prescription would be an improvement.—W.

**Bossu's Stronger Laxative Mixture.**—

Resin of scammony.......................
Resin of jalap...............................aa ½ grain.
Croton oil........................................ 2 drops.
White sugar...............................15 grains.
Mucilage....................................25 grains.
Orange flower water..................... 4 ℈
Compound syrup of senna.............. 1 fl. ʒ
Peppermint water........................ 3 fl. ℥

The first five ingredients having been properly triturated together and emulsionized, the remainder is added carefully. Dose, one teaspoonful.—*New Remedies.*

**For Vaginitis.**—

℞ Tinc. iodinii.................................... ℥ iij

Acidi tanici...................................... ʒ i

Potassii iodidi.................................. ʒ ss M.

Used to paint the vagina in acute or chronic vaginitis, and the uterine neck, in engorgement and ulceration. The proportion of the tinc. of iodine is to be lessened according to the character of the inflamed tissues, and the effect that it is desired to produce.

The same preparation is useful in enlarged tonsils.

**Grisolle's Pills, for Incontinence of Urine.**—

Ext. nucis vom. alcoh...............0.25 gm. ( 4 grains.)
Ferri phosphat (protoph.)...........3.00 gm. (46 grains.)
Ext. quassiæ................................2.00 gm. (31 grains.)
Rad. gentianæ, q. s.
M. 25 pills.

One pill three times a day in conjunction with cold hip-bath, and abstention from drink during the evening.—*New Remedies.*

**Dr. Henry's Cosmeticum.**—For scalp diseases and application for the hair: Spirit, 180 parts; oil of lemon, 3 parts; oil of bergamot, oil of rosemary, and oil of lavender, of each 1 part.—*Hager.*

